# Acute Pericarditis Associated With Graves' Disease: A Case Report

**DOI:** 10.7759/cureus.78588

**Published:** 2025-02-05

**Authors:** Mariana Estrela Santos, Mariana Baptista, Jorge Reis, Raquel Moura, Janine Resende

**Affiliations:** 1 Internal Medicine, Unidade Local de Saúde Gaia Espinho, Vila Nova de Gaia, PRT

**Keywords:** acute pericarditis, autoimmune inflammation, graves' disease, hyperthyroidism complications, thyroid dysfunction

## Abstract

Acute pericarditis is an uncommon but significant complication of Graves' disease. This case report describes a 53-year-old female presenting with acute pericarditis in the context of newly diagnosed Graves' disease. The patient's clinical presentation, diagnostic evaluation, treatment course, and outcome are discussed. We also provide a comprehensive review of the literature on pericarditis associated with Graves' disease, including its pathophysiology, clinical features, diagnostic challenges, and therapeutic approaches. This report highlights the importance of considering thyroid dysfunction in cases of idiopathic pericarditis, as prompt diagnosis and targeted treatment can significantly improve outcomes.

## Introduction

Graves' disease, an autoimmune hyperthyroid disorder, is a leading cause of hyperthyroidism worldwide, with a prevalence of approximately 1.5% in women and 0.5% in men [1,2]. While its most common manifestations include ophthalmopathy, dermopathy, and thyroid dysfunction, it can also present with less typical complications such as acute pericarditis [3]. Acute pericarditis associated with Graves' disease is rare, with only a limited number of cases reported in the literature [4].

Graves' disease, an autoimmune hyperthyroid disorder, is a leading cause of hyperthyroidism worldwide, with a prevalence of approximately 1.5% in women and 0.5% in men [1,2]. While its most common manifestations include ophthalmopathy, dermopathy, and thyroid dysfunction, it can also present with less typical complications such as acute pericarditis [3]. Acute pericarditis associated with Graves' disease is rare, with only a limited number of cases reported in the literature [4].

Although the precise pathophysiologic links between Graves' disease and acute pericarditis remain under investigation, a mounting body of evidence suggests an immune-mediated process underpins this association. In Graves' disease, heightened autoimmune activity - most notably the production of thyroid-stimulating immunoglobulins (TSI) directed against the TSH receptor - along with other antibodies such as anti-thyroid peroxidase (anti-TPO), can trigger widespread inflammatory cascades that extend beyond the thyroid gland [5]. The hypermetabolic state characteristic of hyperthyroidism may exacerbate this inflammation by increasing cardiac output and tissue oxygen demands, potentially contributing to pericardial irritation [6]. When evaluating acute pericarditis in a patient with hyperthyroidism, it is essential to exclude more common etiologies such as viral infections (e.g., coxsackievirus) and other autoimmune disorders (e.g., systemic lupus erythematosus).

Furthermore, social determinants of health (SDOH) - encompassing socioeconomic status, healthcare access, health literacy, and geographic location - can profoundly influence both the diagnosis and management of acute pericarditis. Patients who face financial or logistical barriers to care may experience delays in obtaining timely investigations, specialty referrals, or appropriate medications, leading to suboptimal outcomes. Recognizing and proactively addressing these factors is crucial in delivering equitable care, ensuring early intervention, and ultimately improving the prognosis for individuals with Graves' disease-related pericarditis.

Despite its rarity, recognizing this association is crucial, as timely intervention can lead to excellent clinical outcomes [7].

## Case presentation

A case of a 53-year-old female with no significant past medical history, except for thyroid scintigraphy performed a few weeks prior that suggested a mild goiter and increased technetium uptake, indicative of glandular hyperfunction. This scintigraphy was performed because the patient presented with a six-month history of weight loss (17% of total body index), asthenia, palpitations, heat intolerance, nocturnal hyperhidrosis, and dry throat. The patient had also undergone laboratory testing requested by her attending physician but was still awaiting the results. She had not undergone further evaluation at the time.

Two weeks prior to admission, she developed anterior chest pain exacerbated by inspiration and relieved by leaning forward, as well as progressively worsening dyspnea. Fever had appeared one week before admission and responded to paracetamol. She denied other symptoms or epidemiological exposures, like flu-like symptoms, sore throat, or cough, and that the oropharyngeal exam was normal.

On admission, her vital signs revealed blood pressure of 109/61 mmHg, oxygen saturation of 97% on room air, and a temperature of 38°C. Cardiac auscultation revealed muffled heart sounds, though S1 and S2 were present. The patient remained normocardiac, with no evidence of pulsus paradoxus or jugular venous distention. Beck's triad was absent, indicating no signs of cardiac tamponade.

Laboratory investigations (Table 1) showed an elevated C-reactive protein (15.2 mg/dL) and erythrocyte sedimentation rate (62 mm/h), findings consistent with inflammatory activity.

**Table 1:** Laboratory results EBV - Epstein-Barr virus; CMV - cytomegalovirus; HSV - herpes simplex virus; HIV - human immunodeficiency virus; T4L - free thyroxine

Parameter	Result	Approximate reference range	Interpretation/ comment
Thyroid-stimulating hormone (admission)	< 0.008 µIU/mL	0.4–4.0 µIU/mL	Suppressed, indicating hyperthyroidism
T4L (admission)	2.3 ng/dL	0.8–1.8 ng/dL	Elevated, consistent with hyperthyroidism
Thyrotropin receptor antibodies	5.3 IU/L	< 1.75–2.0 IU/L	Positive, supporting diagnosis of Graves' disease
C-reactive protein	15.2 mg/dL	< 5.0 mg/dL	Elevated, indicative of inflammatory activity
Erythrocyte sedimentation rate	62 mm/h	< 20–30 mm/h (adult female)	Elevated, suggesting significant inflammation
D-dimer	1,964 ng/mL	< 500 ng/mL	Elevated, nonspecific; prompted further imaging to rule out pulmonary embolism
High-sensitivity troponin	Within normal limits	Varies by assay	No evidence of myocardial injury
Infectious Serologies (EBV, CMV, HSV, HIV, etc.)	Negative	Negative	Ruled out infectious etiologies of pericarditis
*Mycoplasma pneumoniae* serology	Negative	Negative	Further excludes common infectious causes
Antinuclear antibodies	Negative	Negative	Decreased likelihood of autoimmune conditions (e.g., lupus)
Anti-double-stranded DNA	Negative	Negative	Decreased likelihood of autoimmune conditions (e.g., lupus)
Follow-up thyroid-stimulating hormone (at 6 months)	3.5 µIU/mL	0.4–4.0 µIU/mL	Returned to normal range after treatment
Follow-up T4L (at 6 months)	1.3 ng/dL	0.8–1.8 ng/dL	Normalized, indicating good control of thyroid function

**Figure 1:**
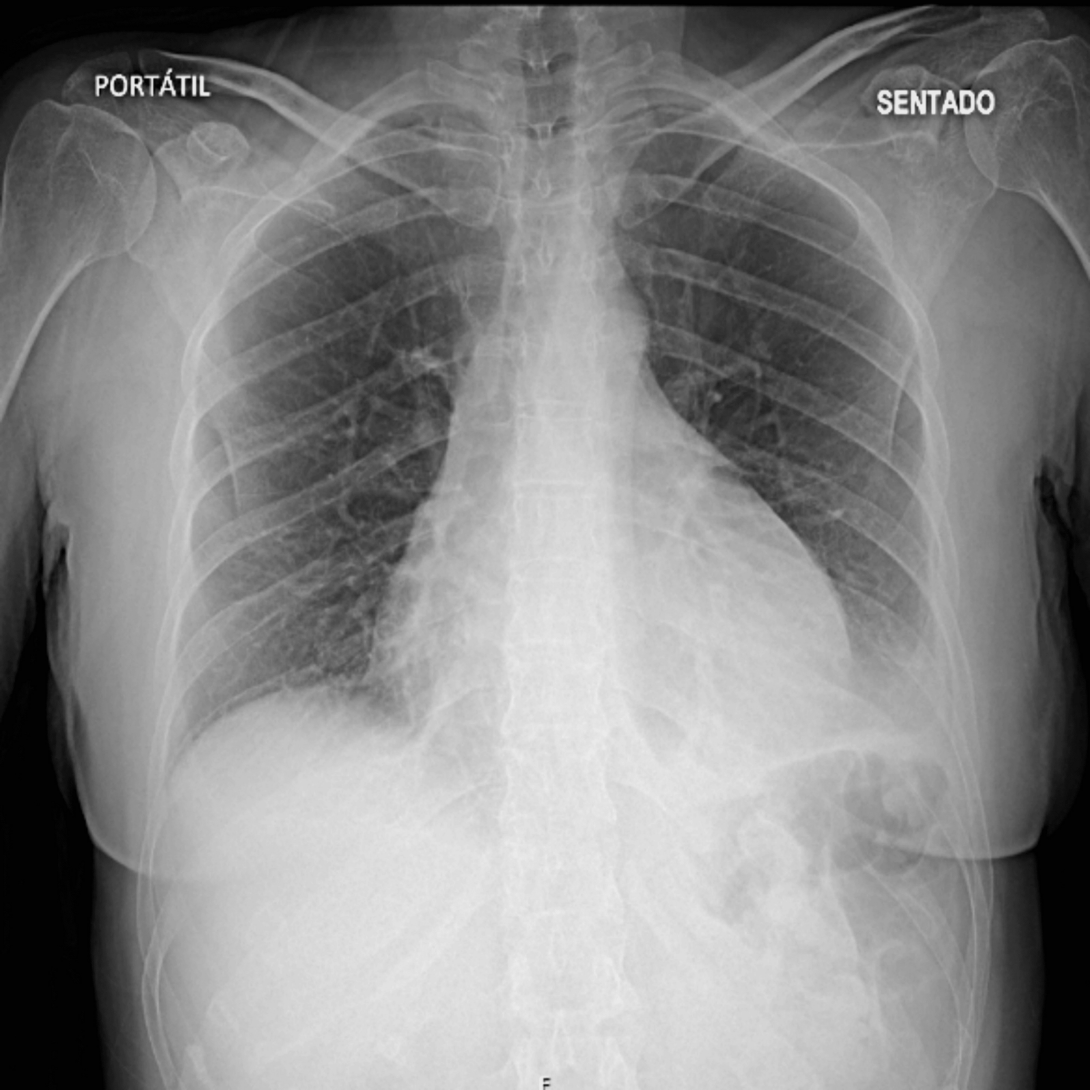
Chest X-ray showing an increased cardiothoracic ratio

**Figure 2:**
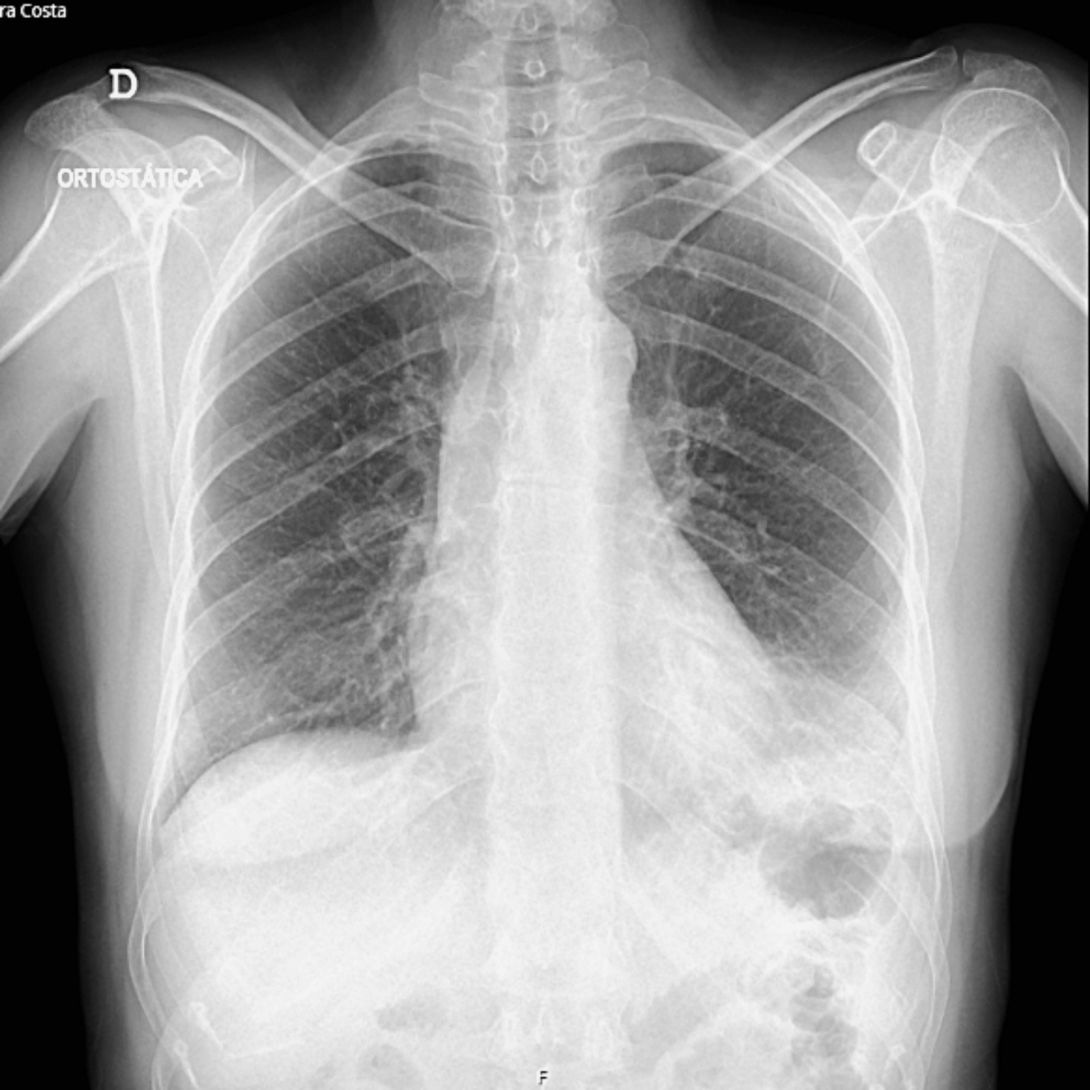
Chest X-ray performed one week earlier

A chest X-ray was performed, revealing an increased cardiothoracic index compared to a previous chest X-ray obtained seven days earlier, which showed no abnormalities (Figures 1, 2).

Although the patient had no known risk factors for pulmonary embolism, given her chest pain at admission and dyspnea, D-dimer testing was requested to exclude PE. The results were elevated (1,964 ng/mL) but nonspecific, thus not confirming the diagnosis; high-sensitivity troponin levels were within normal limits. Consequently, a thoracic CT angiography was performed, which excluded pulmonary embolism but confirmed the pericardial effusion.

**Figure 3:**
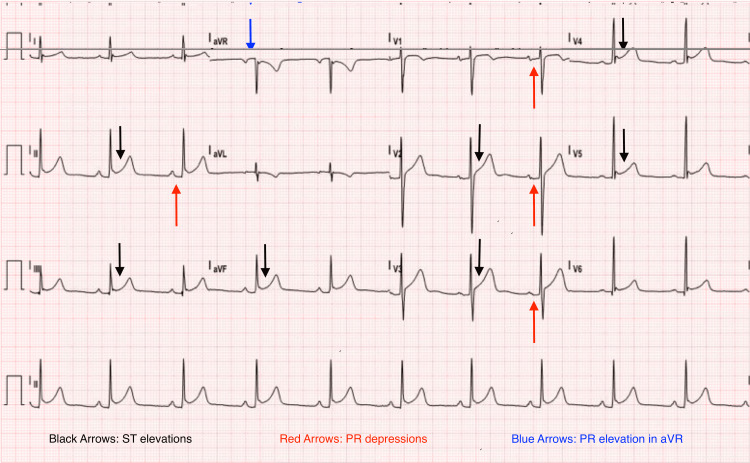
Electrocardiogram revealed generalized concave ST-segment elevation

An electrocardiogram revealed generalized concave ST-segment elevation (Figure 3), and echocardiography demonstrated a circumferential pericardial effusion measuring 1.9 cm, moderate, without signs of tamponade.

Further evaluation (Table 1) revealed suppressed thyroid-stimulating hormone (TSH; <0.008 µIU/mL), elevated free thyroxine (T4L; 2.3 ng/dL), and positive thyrotropin receptor antibodies (TRAb; 5.3 IU/L). Extensive infectious and autoimmune workup, including serologies for Epstein-Barr virus, cytomegalovirus, herpes simplex, human immunodeficiency virus, and *Mycoplasma pneumoniae*, as well as antinuclear antibody and anti-double stranded DNA, were negative. In addition to the previously described negative autoimmune serological markers (antinuclear antibodies and anti-double-stranded DNA), the patient exhibited no other systemic symptoms beyond those reported, effectively ruling out other autoimmune conditions, such as systemic lupus erythematosus, as a cause of the pericarditis. Imaging studies, including abdominopelvic computerized tomography and mammography, excluded malignancy.

Based on clinical findings and the exclusion of other causes, a diagnosis of acute pericarditis associated with Graves' disease was established. The patient was treated with colchicine (0.5 mg for three months), ibuprofen in a tapering regimen (first two weeks 600 mg three times daily; third week 400 mg three times daily; fourth week 400 mg twice daily; and fifth week 400 mg once daily and then stop) and methimazole (initial dose of 20mg/daily with posterior reduction for 10mg/daily two weeks later) for hyperthyroidism. She was discharged two weeks later with complete resolution of symptoms (resolution of chest pain, dyspnea, and fever). At six-month follow-up, echocardiography showed resolution of the pericardial effusion, and thyroid function was stable, with TSH (3.5 µIU/mL) and T4L (1.3 ng/dL). There was no recurrence of pericarditis.

## Discussion

Pericarditis is an uncommon manifestation of Graves' disease, and its pathogenesis remains incompletely understood [8]. Several mechanisms have been proposed: (1) autoimmune inflammation mediated by thyroid autoantibodies (e.g., thyroid-stimulating immunoglobulins (TSI), anti-thyroid peroxidase (anti-TPO)), (2) direct effects of thyrotoxicosis on cardiac and pericardial tissues, and (3) increased capillary permeability [9]. Elevated levels of thyroid hormones may alter the systemic immune response, intensifying the inflammatory milieu around the pericardium. Simultaneously, TSI and other autoantibodies may contribute to local inflammatory cascades. Increased capillary permeability is a phenomenon that can result from both hyperthyroidism-induced hemodynamic changes (e.g., increased cardiac output and blood flow) and immune-mediated endothelial dysfunction, though the exact contribution of each mechanism remains under investigation.

Despite the lack of robust epidemiological data, fewer than 30 cases of Graves' disease-associated pericarditis have been described in the literature, underscoring its rarity [10]. The clinical presentation-chest pain, dyspnea, and fever-often overlaps with other causes of pericarditis (e.g., viral, autoimmune, malignant). However, concurrent hyperthyroid symptoms, such as weight loss, palpitations, and heat intolerance, may prompt suspicion of an endocrine etiology.

A stepwise algorithm is crucial for accurate diagnosis. Suspected pericarditis based on typical symptoms should be investigated with an electrocardiogram (ECG) and inflammatory markers (e.g., C-reactive protein, erythrocyte sedimentation rate). Echocardiography is then performed to confirm pericardial effusion and assess for tamponade physiology. If there are clinical or historical clues suggestive of hyperthyroidism (as in our case), thyroid function tests (TSH, free T4, ± free T3) and autoantibody assays (TRAb, anti-TPO) help determine whether Graves' disease is the underlying cause.

In the present case, the absence of viral serologies, normal imaging to exclude malignancy, and negative autoimmune markers (e.g., antinuclear antibodies, anti-dsDNA) ruled out other potential etiologies, making Graves' disease the most likely cause.

Therapy targets both the pericardial inflammation and the hyperthyroid state. First-line treatment for acute pericarditis typically includes nonsteroidal anti-inflammatory drugs (NSAIDs) and colchicine, which reduce inflammatory activity in the pericardium. In some instances-particularly severe, refractory cases or thyroid storm-corticosteroids may be considered. In our patient, steroids were not used because she responded well to NSAIDs and colchicine, and there was no evidence of thyroid storm or refractory inflammation.

For hyperthyroidism, anti-thyroid drugs such as methimazole or propylthiouracil are essential for restoring euthyroidism. Dose selection depends on the severity of hyperthyroidism; close monitoring of thyroid function guides subsequent dose adjustments. Achieving a stable euthyroid state may reduce or eliminate the inflammatory triggers thought to contribute to pericardial involvement. In our case, there was no need to escalate or modify anti-thyroid therapy specifically for pericarditis once the patient's thyroid function began to normalize and pericardial symptoms resolved.

Although large-scale studies are lacking, existing reports suggest that clinical outcomes in Graves' disease-associated pericarditis are generally favorable, with most patients experiencing complete symptom resolution after appropriate therapy [10]. Case series indicate low recurrence rates, especially once euthyroidism is achieved, though the exact recurrence risk remains uncertain due to the limited number of published cases. No mortality attributable to pericarditis has been reported in these case studies, and most patients recover fully when diagnosis and management are prompt. Future research is needed to better quantify recurrence risk and to determine whether the resolution of hyperthyroidism completely prevents pericarditis relapse.

## Conclusions

This case underscores the rare but clinically relevant association between acute pericarditis and Graves' disease, with fewer than 30 reported cases in the literature to date. The possibility of underdiagnosis suggests that the actual prevalence may be higher. In patients presenting with idiopathic pericarditis - particularly those lacking a viral prodrome or exhibiting signs of hyperthyroidism such as weight loss, palpitations, and heat intolerance - clinicians should maintain a high index of suspicion for underlying thyroid dysfunction. Identifying Graves' disease early may aid in guiding targeted management and optimizing treatment strategies. However, current evidence is limited regarding whether prompt recognition definitively reduces pericarditis-related complications. Further research is warranted to clarify the impact of early diagnosis on clinical outcomes, although treating the hyperthyroid state remains essential for improving overall patient prognosis.

## References

[1] Smith TJ, Hegedüs L (2016). Graves' disease. N Engl J Med.

[2] Bahn RS (2010). Graves' ophthalmopathy. N Engl J Med.

[3] Cooper DS (2003). Hyperthyroidism. Lancet.

[4] Siu CW, Zhang XH, Yung C (2005). Acute pericarditis as a complication of hyperthyroidism: case report and literature review. Heart Vessels.

[5] Bartalena L, Fatourechi V (2014). Extrathyroidal manifestations of Graves' disease: a 2014 update. J Endocrinol Invest.

[6] Mahfoud F, Lüscher TF, Andersson B (2013). Expert consensus document from the European Society of Cardiology on catheter-based renal denervation. Eur Heart J.

[7] Imazio M, Gaita F (2015). Diagnosis and treatment of pericarditis. Heart.

[8] Bozkurt B, Colvin M, Cook J (2016). Current diagnostic and treatment strategies for specific dilated cardiomyopathies: a scientific statement from the American Heart Association. Circulation.

[9] Shetty S, Bhave M, Ghosh K (2011). Acquired hemophilia a: diagnosis, aetiology, clinical spectrum and treatment options. Autoimmun Rev.

[10] Sabatine MS (2021). Approach to the patient with pericardial disease. Braunwald's Heart Disease: A Textbook of Cardiovascular Medicine.

